# Heterotrimeric G-proteins: multi-dimensional regulation in plant growth, development and abiotic stress responses

**DOI:** 10.1007/s44154-024-00188-4

**Published:** 2025-01-06

**Authors:** Shiyuan Guo, Yingge Wang, Jiayan Wu, Xiani Zhou, Huiling Gao

**Affiliations:** National Key Laboratory of Crop Improvement for Stress Tolerance and Production, College of Life Sciences, Northwest A&F University, Yangling, Shaanxi 712100 People’s Republic of China

**Keywords:** Heterotrimeric G-proteins, Molecular switch, Signaling transduction, Stress response

## Abstract

Heterotrimeric G-proteins, comprising Gα, Gβ, and Gγ subunits, act as crucial molecular switches for signaling transduction in all eukaryotic organisms. Through precise modulation of specific receptors or effectors coupled with heterotrimeric G-proteins in signaling cascades, plants have the capability to activate or suppress unique signaling pathways necessary for plant growth, development, and stress responses. This review provides an overview of the heterotrimeric G-proteins signaling pathway obtained to date, and highlights novel areas for future exploration and agricultural application based on the emerging significance and potential of heterotrimeric G proteins in regulating plant development and responses to abiotic stress.

## Introduction

The intricate process of cell signal transduction involves a set of conserved intracellular proteins that transition between active and inactive states to initiate the amplification of signal transduction cascades. These proteins can be categorized into three main types. (i) GTP-binding proteins, comprising heterotrimeric G-proteins (G-proteins) and monomeric G-proteins, modulate the activities of specific effectors when activated by GTP-binding, but are inactivated by GDP-binding (Oldham and Hamm 2008; Ma et al. 2015b; Liu et al. 2021; Mohanasundaram and Pandey 2023); (ii) protein kinases and protein phosphatases (Perraki et al. 2018; Zhang et al. 2023b); (iii) calmodulin (Luan and Wang 2021; Wang et al. 2023). The role of heterotrimeric G proteins in signaling transduction has attracted significant attention in studies of plant development and defense in recent years.

**Fig. 1:**
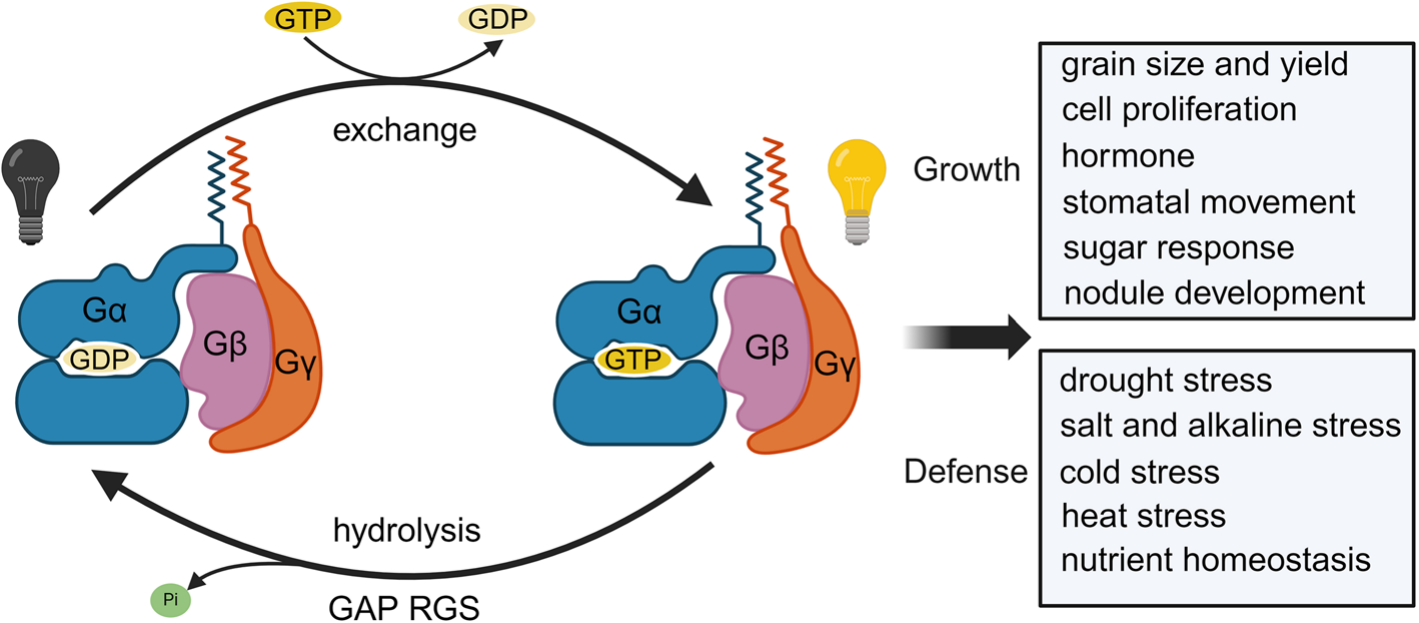
Heterotrimeric G-protein signaling cycle in plant adaptation. In the resting state, Gα keeps it in a GDP-bound state through the GAP activity; In the active state, nucleotide exchange on Gα, resulting in activated GTP-bound Gα and released Gβγ, thus modulating plant growth and defense processes

The membrane-bound heterotrimeric G-proteins are comprised of three conserved subunits: Gα, Gβ, and Gγ (Urano et al. 2013). The Arabidopsis and rice genome encode one canonical Gα member (GPA1/RGA1) and three atypical Gα-like members (XLGs), one Gβ member (AGB1/RGB1), and different types of Gγ members (AGG1/AGG2/AGG3 in Arabidopsis, RGG1/RGG2/GS3/DEP1/qPE9-1/GGC2 in rice) (Urano et al. 2016; Pandey and Vijayakumar 2018; Zhang et al. 2021b). Besides, the regulator of G-protein signaling (RGS) proteins with seven-transmembrane structure that have GTPase-accelerating activity and thus desensitizes the G-protein-mediated signaling (Chen and Jones 2004; Johnston et al. 2007a; Jones et al. 2011b). G-proteins function as molecular switches, playing crucial roles in multiple developmental and defense pathways. These include cell proliferation and division, root and leaf development, stomatal density development, grain size, response to sugar signals, nitrogen and phosphorus-use efficiency, various hormone signals, innate immunity, as well as responses to various abiotic stresses such as cold, drought, salt, and alkaline conditions (Fig. 1) (Sun et al. 2014; Zhang et al. 2021b, 2023a; Tiwari and Bisht 2022; Wang et al. 2024).

## The heterotrimeric G-protein signaling cycle in mammals and plants

In mammal cells, the G protein exists as an inactive heterotrimer consisting of the α subunit (Gα), bound to guanosine diphosphate (GDP), and the βγ dimer (Gβγ) in the absence of a ligand. Binding of the ligand to the G protein-coupled receptors (GPCRs), which acts as a guanine nucleotide exchange factor (GEF), promotes the exchange of GDP for GTP on Gα, thereby activating Gα. The active Gα dissociates from the Gβγ dimer, and both of them can independently interact with and modulate the activity of various downstream effectors (Oldham and Hamm 2008; Milligan and Kostenis 2009). Active Gα has intrinsic GTPase activity, which allows it to hydrolyze GTP back to GDP. This hydrolysis causes Gα to revert to its inactive state and reassociate with Gβγ to reform the inactive heterotrimeric complex, completing the cycle (Urano et al. 2013). Unlike mammals, plant G proteins can active themselves independent of GPCRs. For instance, Arabidopsis G protein (AtGPA1) can spontaneously replace the GDP with GTP to active itself (Johnston et al. 2007a; Jones et al. 2011b; Urano and Jones 2014). Gα intrinsic GTP hydrolysis requires the assistance of GTPase activity-accelerating proteins (GAPs), such as the G protein signaling regulatory protein (RGS) and specific phospholipase C (PLC). The RGS1 which has an N-terminal seven transmembrane (7 TM) domain and a C-terminal cytoplasmic RGS domain promote the GTP hydrolysis of AtGPA1 to keep steady state (Johnston et al. 2007a; Jones et al. 2011a). Signal perception can influence the interaction between AtGPA1 and AtRGS1 that lead to the decoupling of AtGPA1 from AtRGS1. Once AtGPA1 is released from AtRGS1, it becomes less efficient at hydrolyzing GTP. This reduced efficiency helps maintain AtGPA1 in its active state, enabling it to continue modulating the activities of downstream effectors (Fig. 1) (Urano et al. 2012).

## Heterotrimeric G-proteins signaling in plant growth and development

### G-proteins in the regulation of grain size and yield

It is estimated that the world’s population will increase to 9.7 billion by 2050, posing challenges to food production(Ferrero-Serrano and Chakravorty 2023).

Firstly, G-proteins have garnered significant attention in regulating grain size and yield (Xiong et al. 2024). The Gα subunit plays a key role in the development of normal internodes and seeds, as evidenced by the manifestation of morphological abnormalities, such as short internodes and small seeds, upon the suppression of *OsRGA1* in rice (Fujisawa et al. 1999). In particular, the Gγ members (AGG3 in Arabidopsis, GS3/DEP1/qPE9-1/GGC2 in rice, wheat, and barley) play crucial roles in grain size and yield. Additionally, rice DEP1 and GGC2 may have a positive impact on promoting grain size. Additionally, GS3 functions to counteract the effects of DEP1 and GGC2 in order to maintain seed fitness (Fan et al. 2006; Huang et al. 2009; Mao et al. 2010; Li et al. 2012; Wu et al. 2016; Vavilova et al. 2017; Kaur et al. 2018; Sun et al. 2018; Xu et al. 2019). These highly conserved Gγ proteins hold promise as genetic tools for manipulating grain size and yield in important crops such as rice, wheat, and maize. Future challenges and opportunities in G-protein signaling include elucidating the upstream receptors and downstream effectors.

### G-proteins in the regulation of cell proliferation

Secondly, G-proteins play a role in cell proliferation and division processes, thus contributing to crop improvement. For example, Arabidopsis GPA1 positively modulates cell proliferation. *GPA1* mutation seedlings have reduced cell division in aerial tissues (Ullah et al. 2001). In addition, AGB1 acts downstream of GPA1 to inhibit lateral root formation (Chen et al. 2006a). While Arabidopsis AGB1 control stem cell proliferation through interaction with CLAVATA (CLV3) receptor (Ishida et al. 2014). Besides, Arabidopsis AGG3 contributes positively to cell proliferation to control organ size and shape (Li et al. 2012). Significant progress have identified the maize Gα protein COMPACT PLANT2 (CT2) function with CLV receptor to regulate meristem development in maize (Wu et al. 2018). Constitutively active CT2 plants increased higher spikelet density and kernel row number, larger ear inflorescence meristems (IMs) in maize (Bommert et al. 2013; Urano et al. 2015; Je et al. 2018; Springer et al. 2018). Additionally, the loss of RGS1 increased the activity of the Arabidopsis Gα subunit, which manifests as altered cell elongation and proliferation (Chen et al. 2003, 2006a). Hence, G-proteins have significant potential to enhance agronomic traits by advancing genetic technology to meet the needs of the increasing world population.

### G-proteins in modulation of multiple plant hormones

Thirdly, G-proteins signaling play a vital role in controlling plant growth and development, and stress responses through the modulation of multiple plant hormones, including abscisic acid (ABA), auxin, brassinosteroid (BR), ethylene (ET), gibberellic acid (GA), and cytokinin (CK). For instance, GA signaling was partially impaired in the rice dwarf mutant *d1*, which is defective in the Gα subunit (Ueguchi-Tanaka et al. 2000). In Arabidopsis, G protein-coupled receptor GCR1 may negatively regulate the GPA1-mediated ABA responses in guard cells. The *gcr1* mutant exhibits hypersensitivity to ABA, with improved drought tolerance and lower rate of water loss, while *gpa1* mutant shows insensitivity (Pandey and Assmann 2004). Studies demonstrate that AGB1 mainly regulates auxin transport through interaction with N-MYC DOWNREGULATED-LIKE1 (NDL1) (Ullah et al. 2003; Mudgil et al. 2009). Adaptor protein AP-3µ interacted with AGB1 positively regulates the ABA responses in seed germination development (Kansup et al. 2013). Besides, AGB1 modulates BR signaling in a BES1-dependent manner (Zhang et al. 2018). EXTRA-LARGE G PROTEINs (XLGs) and PUB2/PUB4 E3 ligases regulate cytokinin responses and developmental processes (Wang et al. 2017). In tomato, the type B Gγ subunit SiGGB1 mediates auxin and ABA signaling (Subramaniam et al. 2016).

### G-proteins in the regulation of nodule formation

In addition, studies have uncovered that the phosphorylation-dependent regulation of G-proteins and Regulator of G-protein Signaling (RGS) during nodule formation in Soybean (Choudhury and Pandey 2013, 2015; Roy Choudhury and Pandey 2022). The active Gα subunit and RGS play negative and positive roles in nodule formation respectively. Here, the RGS is phosphorylated by Nod factor receptor (NFR1) through physically interaction, which keep the inactive state of Gα subunit, thus promoting successful nodule development (Choudhury and Pandey 2015). Recent study shows symbiosis receptor kinase (SymRKα/β or NORK) could interact with and phosphorylate Gα in vitro, overexpression of phosphor-mimetic Gα subunit significantly increases nodule number in soybean (Roy Choudhury and Pandey 2022).

### G-proteins in the regulation of stomatal movement

Besides, G-protein signaling exhibit essential roles in guard cell development and movement. Stomatal opening in *gpa1* or *agg3* mutant are insensitive to inhibition by ABA, with higher rate of water loss (Wang et al. 2001; Chakravorty et al. 2011). Further, researchers reveal that AGB1 is required for the Rapid Alkalinization Factor (RALF1) regulation of stomatal movement through interaction with the receptor-like kinase FERONIA (FER) (Yu et al. 2018).

### G-proteins in sugar signaling

Sugars are signal molecules that play a fundamental role in regulating plant growth, development, and stress responses. Finally, emerging evidence suggest that G-protein pathway participate in sugar signaling (Ullah et al. 2002). For instance, the *gpa1* null mutant seedlings exhibit sensitive phenotype, but the overexpressing GPA1^(Q222L)^ show more tolerant to high D-glucose treatment. It is reported the Plastid Protein THYLAKOID FORMATION1 (THF1) physically interact with GPA1, thus involved in sugar signal transduction between plastids and plasma membrane (Huang et al. 2006; Johnston et al. 2007b). Further, biochemical and genetic evidence confirm that BR receptors BRI1 and BAK1 physically interact with and phosphorylate G proteins to modulate sugar responsive growth and development (Peng et al. 2018). RGS1 also plays a vital role in sugar signaling. D-glucose causes RGS1 endocytosis, leaving the Gα constitutively active for sugar signaling. The *rgs1-2* mutant is low sensitive to Glc during seed germination (Chen 2006; Johnston et al. 2007a; Urano et al. 2012). These discoveries shed light on the intricate interplay between the heterotrimeric G-proteins and plant growth and development processes, providing new insight into how plants sense multiple environmental cues to fine-tune growth and development via G-protein-coupled signaling pathway (Table 1).

**Table 1:** Summary of essential roles of G-proteins in plant growth and development

Functions of G-proteins	G-protein subunit &phenotype	References
Grain size and yield	Gα: suppression of *OsRGA1* exhibited morphological abnormalities, such as short internodes and small seeds in rice. Gγ: gain-of-function of *OsDEP1* and *OsGGC2* had increased number of grains per panicle and higher grain yield; loss-of-function of *OsGS3* resulted in longer grain size; overexpression of *AGG3* showed increased seed size and seed number per plant in *Camelina sativa*	(Fujisawa et al. 1999; Fan et al. 2006; Huang et al. 2009; Mao et al. 2010; Li et al. 2012; Wu et al. 2016; Vavilova et al. 2017; Kaur et al. 2018; Sun et al. 2018; Xu et al. 2019; Xiong et al. 2024)
Hormone	Gα: *atgpa1* mutant exhibited insensitivity to ABA response in Arabidopsis; *d1* mutant affected GA signal transduction in rice; Gβ: mutant plants were hypersensitive to auxin and ABA; *agb1* mutants displayed BR hyposensitivity; loss-of-function of *AGB1* was hypersensitive to ethylene; Gγ: *SlGGB1* silenced lines were hypersensitive to exogenous auxin and hyposensitive to ABA in tomato. XLG: loss-of-function of *XLG3* was hypersensitive to ethylene; *xlg1/2/3* triple mutants exhibited defects in cytokinin responses	(Ueguchi-Tanaka et al. 2000; Ullah et al. 2003; Pandey and Assmann 2004; Mudgil et al. 2009; Kansup et al. 2013; Subramaniam et al. 2016; Wang et al. 2017; Zhang et al. 2018)
Cell proliferation	Gα: *gpa1* null mutants reduced cell division in Arabidopsis with fewer lateral roots; Constitutively active *CT2* plants increased higher spikelet density and kernel row number, larger ear inflorescence meristems (IMs) in maize; Gβ: mutant plants had excessive lateral root and enlarged stem cell region in Arabidopsis; Knockdown of Gβ: genes caused reduced longitudinal and enhanced transverse expansion in *Camelina sativa*	(Ullah et al. 2001; Chen et al. 2006b, a; Bommert et al. 2013; Ishida et al. 2014; Urano et al. 2015; Je et al. 2018; Springer et al. 2018; Wu et al. 2018)
Stomatal movement	Gα: Mutation of *gpa1* resulted in ABA insensitivity of stomatal opening and greater water loss rate in Arabidopsis; Gβ: mutant plants were hyposensitive to ABA inhibition of stomatal opening in Arabidopsis; Gγ: *agg3* mutants were hyposensitive to ABA inhibition of both stomatal opening and guard cell K^+^_in_ currents	(Wang et al. 2001; Chakravorty et al. 2011; Yu et al. 2018)
Sugar response	Gα: mutant plants were hypersensitive to glucose in Arabidopsis; Gβ: mutant plants were hypersensitive to glucose in Arabidopsis	(Ullah et al. 2002; Huang et al. 2006; Johnston et al. 2007a, b; Peng et al. 2018)
Nodule development	Gα: RNAi lines increased nodule number in soybean; Gβ: overexpression of Gβ3 and Gβ4 increased nodule number in soybean; Gγ: overexpression of Gγ4 increased nodule number in soybean	(Choudhury and Pandey 2013, 2015; Roy Choudhury and Pandey 2022)

## Heterotrimeric G-proteins signaling in plant abiotic stress responses

**Fig. 2:**
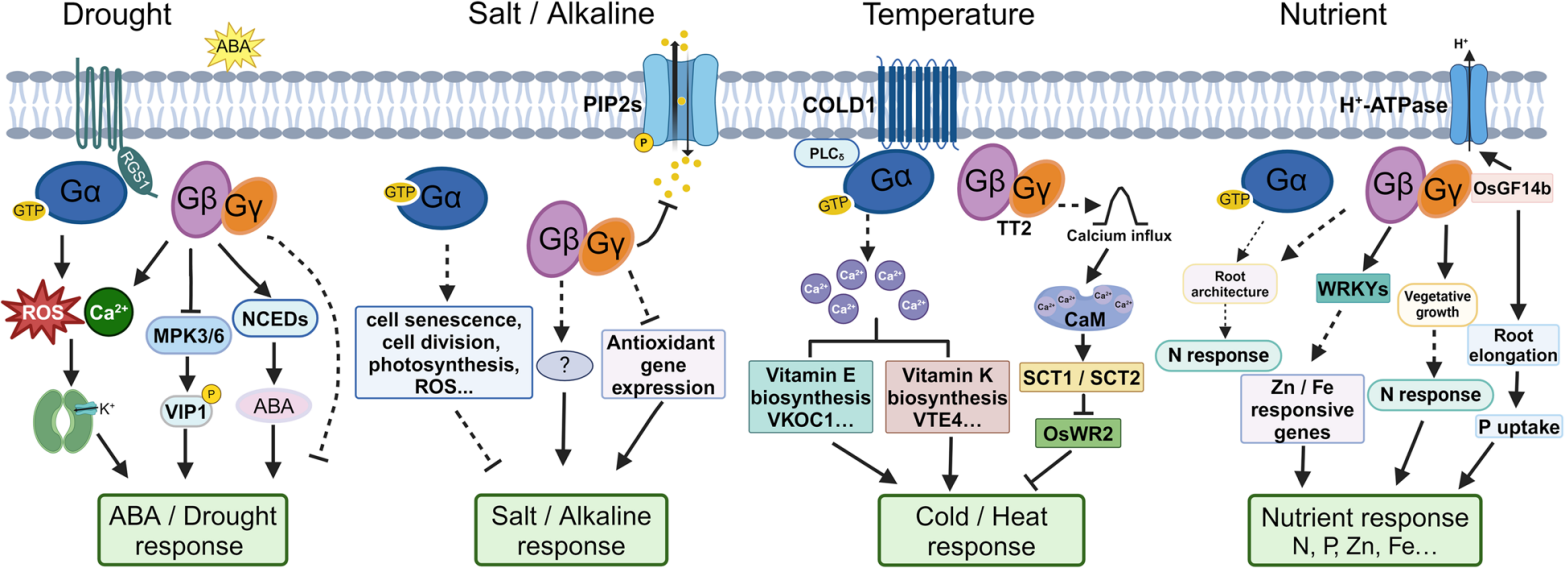
Heterotrimeric G-protein signaling in plant abiotic stresses. (1) Under drought stress, GPA1 function in ABA inhibition of K^+^ influx channels in guard cells. AGB1 negatively modulates MPK3/6-mediated VIP1 phosphorylation or promotes ABA biosynthesis by upregulating NCEDs gene expression. (2) Gα and Gβ negatively and positively regulates salt stress respectively through unknown molecular mechanism. Gγ (AT1) modulates the phosphorylation level of aquaporins PIP2s to protect plant cells against oxidative stress under alkaline stress. (3) COLD1 confers chilling tolerance through interacting with Gα to activate the Ca^2+^ channel by influencing vitamin biosynthesis. Gγ (TT2) facilitates the elevation in cytosolic Ca^2+^ level, and SCT1 binds *OsWR2* promoter to negatively regulate of wax biosynthesis under heat stress. (4) G-protein subunits function in nutrient response, such as N, P, Zn, and Fe. For instance, Gγ (DEP1) is required for nitrogen/phosphate-use efficiency in rice

The environment is becoming increasingly complex and volatile, raising concerns about its potential adverse impact on global crop production and quality. Over the past few decades, substantial advancements have been achieved in understanding various stress signaling pathways, focusing on signal perception, transduction, and downstream responses to both biotic and abiotic stresses (Zhu 2016; Gong et al. 2020; Zhang et al. 2021a; Ma et al. 2024). To ensure their survival and reproduction in dynamic environments, plants leverage a series of molecular mechanisms, including the rapid shifting of G-proteins to transduce extracellular signals into intracellular responses. These stress responses include both abiotic (drought, salinity, alkaline, cold, temperature, heavy metal, and nutrient deficiency) and biotic stresses (fungi, bacteria). These studies have substantially advanced our understanding of the molecular and genetic mechanism underlying plant stress resistance, and opened the era of molecular design breeding of stress-resistant crops. Here, we primarily summarize recent advancements in understanding the molecular functions of G-proteins in abiotic stress responses (Fig. 2).

### Response to drought stress

Drought is a significant limitation to agricultural production in arid and semi-arid regions. Plants have developed diverse strategies to adapt to drought stress, including root water absorption, water storage and utilization, stomatal dynamics, physiology and molecular mechanisms to tolerate short- or long-term responses to drought stress. Among these, the phytohormone abscisic acid (ABA) plays a critical role in addressing environmental stimuli, particularly drought (Gong et al. 2020; Gupta et al. 2020; Zhang et al. 2021a; Muhammad Aslam et al. 2022; He et al. 2024). Studies have demonstrated that G-proteins are involved in drought resistance. Overexpression of RGS1 can strengthen the inhibition of ABA-mediated root elongation and drought tolerance (Chen et al. 2006b). Previous analysis of mutants deficient in Arabidopsis canonical Gα and Gβ subunit, GPA1 and AGB1 antagonistically modulate stomatal density (Zhang et al. 2008; Nilson and Assmann 2010). GPA1, is disrupted in ABA inhibition of K^+^ influx channels and pH-independent ABA activation of anion efflux channels in guard cells. The *gpa1* mutant shows ABA hyposensitive phenotype in stomatal opening, indicating GPA1 acts upstream of ROS and Ca^2+^ channel activation in guard cell signaling network (Zhang et al. 2011). Additionally, in rice, the *RGA1* mutant, *d1*, exhibits reduced sensitivity to drought stress with lower leaf temperature and higher photochemical reflectance, indicating the potential utility of manipulating G-protein signaling for breeding drought-tolerant rice (Ferrero-Serrano and Assmann 2016; Ferrero‐Serrano et al., 2018; Zait et al. 2021).

Previous studies demonstrated that AtAGB1 regulates reproductive trait plasticity in response to water availability (Nilson and Assmann 2009), and it negatively modulates drought tolerance through down-regulating Mitogen-activated protein kinase 6 (AtMPK6), VirE2-interacting protein1 (AtVIP1) and AtMYB44 cascade in Arabidopsis (Zhang et al. 2015b). A recent study identified AGB1 negatively modulates MPK3-mediated VIP1 phosphorylation by outcompeting VIP1 for interaction with MPK3-C terminal in the absence of ABA; While elevated ABA could activate the MPK3-VIP1 cascade which repress AGB1 expression, thus augmenting ABA signaling pathway (Xu et al. 2024). AtAGB1 is also involved in guard cell extracellular calcium (Ca_o_) signaling and calcium-induced calcium release, the stomatal movement of the *agb1* mutant exhibited insensitive to Ca_o_ (Jeon et al. 2019). Proteome analysis showed that AtAGB1 is required to maintain intracellular redox homeostasis coupled with ABA signal (Smythers et al. 2022). In bread wheat, TaGB1, which encodes a Gβ subunit, contributes to drought and salt tolerance, with higher survival rate, SOD and proline content in the TaGB1-B overexpression lines (Xiong et al. 2023).

The atypical Gγ subunit AGG3 has been reported to modulate guard cell K^+^ channel and germination in response to ABA. The *agg3* mutants exhibits hyposensitive to ABA inhibition of stomatal opening and K^+^ current in guard cell (Chakravorty et al. 2011), while the stomatal aperture of *agg1*, *agg2*, or *agg1 agg2* double mutants shows no significant difference compared with WT in response to ABA (Trusov et al. 2008). In rice, the Gβ subunit RGB1 and Gγ subunit qPE9-1 positively and negatively modulate in ABA-dependent drought stress response. RGB1 promotes ABA biosynthesis by upregulating 9-*cis*-epoxycarotenoid dioxygenases (NCEDs) (*OsNCED4* and *OsNCED5*) gene expression, and ABA or drought stress stimulate the RGB1 transcription. While qPE9-1 regulates ABA signaling by suppressing the expression of essential transcription factors (Abscisic acid-insensitive 5 (ABI5), (NAM, ATAF1/2, CUC1/2) transcription factors (NACs)) involved in ABA and stress responses (Zhang et al. 2015a). These discoveries illuminate the intricate interplay between G-protein signaling and drought stress response pathways, offering new insights into how plants adjust their responses to drought stress and maintain growth balance by finely tuning the levels of G-protein signals.

### Response to salt and alkaline stress

Excess soil salinity and alkalinity affect extensive land areas and represent significant constraints on global crop production (Liang et al. 2024). The toxic effects of salt stress on plants occur in a two-phase: an initial osmotic stress phase characterized by rapid water loss, followed by a subsequent ionic stress phase marked by the accumulation of salt ions, predominantly Na^+^ and Cl^−^. Plants have evolved intricate regulatory networks to mitigate these harmful effects, including uptake, transport, compartmentalization of salt ions, antioxidative system, and trade-off between growth and salt-alkali stress (Zhu 2002; Gong et al. 2020; Zhang et al. 2021a). Studies has been made in identifying the potential role of G-proteins underlying the salt tolerance.

The Gα subunit modulates cellular senescence, cell proliferation and division under salt stress. Loss of function mutant of Gα, *DK22* and *CT2*, exhibit reduced growth inhibition and leaf senescence caused by sodium toxicity in rice and maize (Urano et al. 2014). Similarly, a novel allele of Gα subunit *RGA1* mediates salt tolerance partially through ROS scavenging by proteomic analysis. The *sd58* mutant displays enhanced salt resistance and dwarf phenotypes (Peng et al. 2019). These findings demonstrate the heterotrimeric G-proteins could be valuable candidates for the development of salt stress-tolerant crops.

*AGB1* is up-regulated by salt stress, but down-regulated by cold and heat stress in Arabidopsis (Ma et al. 2015a). Compared with WT, the *agb1* mutant confers hypersensitivity to high NaCl treatment, with decreased cotyledon greening rates, fresh weight, root length, survival rate, and obvious chlorotic lesions in older leaves. Whereas the Na^+^/K^+^ concentration and malonaldehyde (MDA) content increase significantly (Colaneri et al. 2014; Ma et al. 2015a). Furthermore, AGB1 regulates root to shoot Na^+^ translocation through the transpiration stream, and the Ca^2+^ ameliorates the salinity sensitivity of *agb1* mutant through modulating ion flux in the root (Yu and Assmann 2015). AGB1 and RLK FERONIA (FER) act synergistically in salt tolerance, the survival rate of *agb1-2 fer2* double mutant shows significantly lower than the single mutant *agb1-2* or *fer2,* which both are involved in salt-induced ROS production (Yu and Assmann 2018). In wheat, Gβ subunit TaGB1-B could improve the drought and salt tolerance. Overexpression of *TaGB1-B* lines exhibits higher survival rate than that of control through enhancing the ability of active oxygen scavenging (Xiong et al. 2023). These findings indicate that AGB1 plays an essential role in balancing growth and the response to salt stress, thus suggesting its potential utility for the molecular design of salt-tolerant, high-yield crops.

Plant G-protein γ subunit RGG1 promotes salt tolerance, probably by increasing the transcript levels of antioxidative genes (*CATa*, *APX1*, *GR2*), thereby elevating detoxification of ROS in response to salt stress in rice. *RGG1* is highly transcriptionally upregulated under salt treatment. Overexpression of RGG1 shows high salinity tolerance even in 200 mM NaCl condition (Swain et al. 2017). However, the exact molecular mechanism of RGG1 in mediating plant salt-stress responses need to be elucidated. To explore the evolutionarily conserved mechanism underlying the plethora and distinctions among G-protein subunits, results illustrate that G-proteins modulate stress readiness through regulating transcriptional and metabolic homeostasis between *Arabidopsis thaliana* and *Marchantia polymorpha*. For instance, WRKYs transcription factors mediate G protein-regulated transcriptional alterations to salt stress, suggesting the variation in degree of stress tolerance in different species and ecotypes may partly be explained by activating G-protein signaling level or other stress regulators (Wu et al. 2022). Recently, notable advances have been illustrated in the discovery of the natural alleles of AT1, an atypical G-protein γ subunit, greatly contribute to alkaline tolerance in sorghum and other three different monocotyledonous crops (rice, maize, and millet). *NIL-Sbat1* (an *at1* allele with a C-terminal truncation) exhibits hypersensitivity, but knockout of *AT1* greatly increased alkaline tolerance in sorghum. Under alkaline stress, AT1 modulates the phosphorylation level of aquaporins PIP2s, thus regulating ROS level to maintain H_2_O_2_ homeostasis, thus protecting plant cells against oxidative stress (Ju and Wang 2023; Zhang et al. 2023a). In addition, *TaAT1* knockout can also greatly enhance salt-alkaline tolerance in wheat with the conserved mechanism as that in sorghum and rice (Sun et al. 2023). These studies demonstrate that the significant potential of G-proteins in modulating salt-alkaline tolerance.

### Response to temperature stress

Environment temperature is a vital factor that affects plant growth, development, and distribution, and crop production. Plants have evolved a set of sophisticated mechanisms to withstand extreme temperature stresses, such as cold and heat stress (Ding et al. 2020; Chen et al. 2022; Kan et al. 2023). In *Pisum sativum*, expression of Gα and Gβ are induced by heat, salt and H_2_O_2_ treatment. Gα/Gβ-overexpressing plants in transgenic tobacco demonstrated improved heat tolerance, with a higher germination rate. This is achieved through the interaction of G-protein and phospholipase C (PLC_δ_), then PLC could activate the GTPase activity of Gα (Misra et al. 2007). The transcript of *RGA1* is also upregulated under abiotic stress including drought, heat, cold and NaCl stresses through whole transcriptome microarray analysis in rice (Yadav et al. 2013; Jangam et al. 2016). Plasma membrane and endoplasmic reticulum (ER) localized COLD1 (CHILLING-TOLERANCE DIVERGENCE1), confers chilling tolerance through interacting with Gα subunit (RGA1) to activate the Ca^2+^ channel for modulating chilling tolerance in *japonica* rice. COLD1 acts as GTPase-accelerating factor to accelerate RGA1 GTPase activity. Overexpression of *COLD1*^*jap*^ exhibited remarkably higher tolerance to chilling, whereas the *cold1-1* mutant, as well as of the antisense transgenic lines showed chilling sensitivity (Ma et al. 2015b; Luo et al. 2021). Rice G-protein γ subunit (*RGG1* and *RGG2*) are upregulated following cold and heat treatment. Also a type C Gγ subunit, CsGG3.2 positively modulates the expression of CBF genes for chilling tolerance through enhancing ROS scavenging in *Cucumis sativus L* (Bai et al. 2018). In addition, rice Gγ subunit TT2 (THERMOTOLERANCE 2), confers thermotolerance. Disrupted *TT2* increased thermotolerance with greater retention of wax upon heat treatment. TT2 facilitates the elevation in cytosolic Ca^2+^ level, and SCT1 (Sensing Ca^2+^ Transcription factor 1) binds *OsWR2* promoter to negatively regulate of wax biosynthesis under heat stress. While loss of function of *TT2* attenuates the heat-triggered transcriptional downregulation of *OsWR2* (Kan et al. 2021)*.* Recently, Histone deacetylase OsHDA716 was reported to repress cold tolerance through interacting with and deacetylating OsbZIP46 transcription factor, thus reducing OsbZIP46 protein stability and transcriptional gene regulation of *COLD1* and *OsDREB1A* (Sun et al. 2024). These results demonstrate that the G-protein signaling underlies a vital mechanism in response to temperature stress, facilitating the molecular design of cold or heat-tolerant crops.

### Response to nutrient stress

Plants require essential or beneficial nutrients to support growth and development throughout their lifecycle, and the efficient use of nutrients has always been a primary focus in the field of plant nutrition, which directly affect agricultural productivity and food security (Gong et al. 2020; Brown et al. 2021; Kiri 2023). Significantly, progress has been made in exploring the intricate mechanisms that modulate nutrient uptake, translocation, and utilization in plants. Emerging evidence demonstrate that G-proteins play significant roles in plant nutrient homeostasis, including nitrogen(N), phosphate (P), Zinc (Zn), and Iron (Fe) (Sun et al. 2014; Takahashi et al. 2020; Wang et al. 2021).

Transcriptome analysis shows that GPA1 and AGB1 modulate multiple processes, including biotic and abiotic stresses, including nutrient response (N, P, Zn and Fe) (Chakraborty et al. 2015; Takahashi et al. 2020). For instances, root architecture of *dk22* (Gα subunit in rice) and *agb1* (Gβ subunit in Arabidopsis) mutants differ in response to different nitrogen treatments, with the significantly lower fresh root weight in the *dk22* mutant, and higher lateral root number in the *agb1* mutant (Liang et al. 2018). The *agb1* mutant exhibits sensitive phenotype under Zn stresses, and further WRKY25 or WRKY33 may function downstream of AGB1 to mediate the Zn stress response (Takahashi et al. 2020). Significantly, the Gγ subunit DEP1 is required for nitrogen-use efficiency in rice, and *DEP1* has been subjected to artificial selection during *japonica* rice evolution. The dominant *dep1-1* allele plants exhibit increased nitrogen uptake and grain yield under moderate nitrogen treatment, whereas the constitutive overexpression of *DEP1* shows no obvious phenotype (Sun et al. 2014). In addition, rice *qPE9-1* is transcriptionally upregulated under low P conditions. The rice varieties carrying the qPE9-1 allele exhibit longer primary root length and P concentration than those varieties with the *qpe9-1* allele under low P conditions. Mechanically, qPE9-1 interacts with 14–3-3 protein OsGF14b to increase plasma membrane H^+^-ATPase activity, thus facilitating better root growth (Wang et al. 2021). These results demonstrate that G-protein signaling, characterized as the most critical plant nutrient-related quantitative trait loci (QTL), has highlighted the potential of modulating G-protein pathway to improve future agriculture.

## Conclusions and perspectives

Overall, G-proteins are widely utilized as molecular switches in abiotic stress responses, offering a regulatory mechanism for the rapid adaptation of plants to ever-changing environments. These studies collectively illustrate the essential roles played by G-proteins and their regulators or targets in salt, alkaline, temperature, and nutrient tolerance. However, the underlying molecular mechanism are not yet fully elucidated, including: 1) Despite numerous discoveries regarding plant heterotrimeric G-proteins, research on the structure and function of G-protein subunits remains limited. The molecular mechanisms governing the activation, inactivation, and downstream effector circulation of G-proteins are largely unknown. What is the significance of the C-terminal of type III Gγ containing cysteine? How is the specificity of plant G protein signaling achieved? How G-proteins balance growth, development and stress tolerance? Therefore, efforts and great challenges need to elucidate how plant G-proteins interact with a variety of hormones and environmental signals to maintain growth and development in changing environment, in combination with other biochemistry, structure or systems biology in the future. 2) Identifying downstream effectors of plant G-proteins is a critical step in elucidating their signal transduction mechanisms. At present, the researches focus on the functions of plant G-proteins signaling mainly using various mutants, but their detailed biochemical properties remain to be fully revealed. New technologies need to be used to uncover different targets upstream or downstream of plant G-proteins coupled signaling pathways, including proteomics, TurbolID, and APEX, aiding in the exploration of G proteins-substrate interactions network. Exploration of upstream or downstream effectors not only contributes to our understanding of the fundamental mechanisms of G-protein signaling, but also holds profound implications for applications such as crop improvement, and plant disease control. 3) The reasons for diametrically opposed role of different G-protein subunits or subunits of the same species under different stresses are needed to be further investigated. Especially how do tissues or organelles interconnect and respond to G-protein signaling? 4) Phosphorylation modification plays a crucial role in G-protein signal transduction. The roles of other post-translational modifications, such as methylation, ubiquitination, and SUMOylation remain to be explored. 5) At present, the researches on G-protein are mainly focus on the model plants *Arabidopsis thaliana*, and rice, while exploring the multiple functions of G-proteins in other plants, especially crops, are also worthy. Furthermore, precise editing of G-proteins will provide the strategies to produce ideal crops with high yield and quality in changing environment.

The well-executed studies described above illustrate that distinct G-proteins are capable of activating or deactivating specific signaling pathways in response to extracellular stimuli through different downstream effectors in ever-changing environments. Naturally, candidate proteins could potentially act as receptors or effectors of heterotrimeric G protein signaling have garnered increasing attention in plant biology. Furthermore, candidate effectors downstream of the G protein signaling involved in various stress responses urgently require further investigation and characterization in the future. Recent advancements have demonstrated that AT1/GS1, an atypical Gγ subunit, enhances alkaline tolerance by regulating PIP2 phosphorylation in ROS distribution in sorghum, offering potential for designing crops with significantly improved resistance to high alkaline stress (Zhang et al. 2023a). More in-depth explorations and functional characteristics of the structures, regulatory mechanisms, and functions of G-proteins will contribute to understand their potential diverse roles in plant growth and development, and defense against stimuli. Therefore, precise editing of vital molecular switches, including G-proteins, calcium, and phosphocode, will offer strategies for producing ideal crops with higher yield, quality, and strong defense resistance in changing or extreme environments. Meanwhile, the balance between plant growth and defense response must be carefully considered in genetic modification for crop breeding.

## Data Availability

Not applicable.

## Abbreviations

CLV3: CLAVATA receptor
CT2: COMPACT PLANT2
IMs: Inflorescence meristems
NDL1: N-MYC DOWNREGULATED-LIKE1
XLGs: EXTRA-LARGE G PROTEINs
RGS: Regulator of G-protein Signaling
NFR1: Nod factor receptor
RALF1: Rapid Alkalinization Factor 1
THF1: THYLAKOID FORMATION1
MDA: Malonaldehyde
FER: FERONIA
PLC_δ_: Phospholipase C
COLD1: CHILLING-TOLERANCE DIVERGENCE1
TT2: THERMOTOLERANCE 2
SCT1: Sensing Ca^2+^ Transcription factor 1
QTL: Quantitative trait loci
MPK6: Mitogen-activated protein kinase 6
VIP1: VirE2-interacting protein1
NCEDs: 9-*cis*-Epoxycarotenoid dioxygenases
NACs: NAC (NAM, ATAF1/2, CUC1/2) transcription factors
ABI5: Abscisic acid-insensitive 5
